# Digital Health to Strengthen District-Based Public–Private Mix Tuberculosis Control in Purwakarta District, Indonesia: A Qualitative Research

**DOI:** 10.3390/ijerph191912097

**Published:** 2022-09-24

**Authors:** Cindra Paskaria, Deni Kurniadi Sunjaya, Ida Parwati, Muhammad Begawan Bestari

**Affiliations:** 1 Faculty of Medicine, Maranatha Christian University, Bandung 40164, Indonesia; 2 Doctoral Study Program, Faculty of Medicine, Universitas Padjadjaran, Bandung 40132, Indonesia

**Keywords:** tuberculosis, district-based public–private mix, digital health

## Abstract

This study aimed to explore the problem that occurs in district-based public–private mix tuberculosis (DPPM TB) in the Purwakarta district, and how digital health can help overcome this problem. This study used a qualitative research design. By purposive sampling, 46 informants were selected to be interviewed and 9 informants participated in focus group discussion. Data were collected during January to November 2020 and analyzed using the content analysis technique. Trustworthiness is obtained through the triangulation method and peer debriefing. The problems identified in public and private partnership were the lack of communication and awareness, the under-reporting of TB cases in the private health sector, and the limitation of the existing information system. Communication is important in delivering information about a tuberculosis program, patient referrals, and contact investigation; therefore, digital health is considered as a potential strategy to facilitate that. Digital health must consider ethical issues, avoid redundancy, be user-friendly, and include intervention packages. We conclude that the lack of communication between the public and the private health sectors in TB control was a major problem in DPPM TB. Digital health is needed to ensure the flow of information and communication between the public and the private health sectors.

## 1. Introduction

The epidemic of tuberculosis (TB) is still a world health problem and one of the top ten causes of death. Globally, an estimated 9.9 million (8.9–11.0 million) people fell ill with TB in 2020. Indonesia is ranked as the third highest TB-burden country in the world (8.4%), but more than half of TB cases were not notified in the national TB control program (NTP), and they are called missing cases. Missing cases are TB patients who are unreported, undiagnosed, or do not have access to health facilities. This would impact the spread of TB and increase the mortality [1,2].

Health services for TB patients in Indonesia were carried out by the public (community health centers, public hospitals) and private sectors (private hospitals, clinics, private physicians, private practice midwives). There are some physicians who engage in dual practice, both the public and private health centers [3,4]. Public health care facilities consist of 9601 community health centers (CHCs) and 861 public hospitals. The number of private health care facilities in Indonesia has been increasing. There are 110,000 registered physicians, 1553 private hospitals (approximately 64% of the total number of hospitals in Indonesia), 24,716 pharmacies, and 8615 licensed drug shops [3].

The patient pathway analysis study in Indonesia in 2017 showed that the role of private health services in the diagnosis, treatment, and reporting of TB cases was lower than the public health services, whereas most TB patients seek initial treatment to private health care facilities [5]. Global TB strategy has recommended involving the private health sector in NTP, known as Public–Private Mix (PPM) [6].

Indonesia adopted a decentralized system of government, in which the government consists of central, provincial, and district bodies. The health sector is one of the decentralized sectors. In terms of the health services, the central government is responsible for tertiary referral hospital. The provincial government is responsible for secondary referral hospital. The district government have the responsibility of providing basic health care through CHCs and primary referral hospital [3,7].

The central government establishes the national health policy. The role of the district is in the operationalization of central government policies in its region. The TB program as a vertical program, and the main strategy of the central government was affected by this decentralization context. Thus, in Indonesia, PPM has been implemented under the coordination of the district health office (DHO), so it is called district-based public–private mix (DPPM). DPPM is a collaboration between public and private health sector to find all TB cases and ensure that these patients receive standardized health services until they are cured wherever they choose to seek care [8]. Involvement of the private sector in TB programs can improve TB case detection and notification, as well as reduce delayed diagnosis and treatment, thereby reducing TB patient care cost. Failure to engage the private sector in a TB program leads to increased transmission, excess morbidity and mortality, increasing drug resistance, unnecessary impoverishment, and delayed introduction of improved TB tools [9]. 

TB diagnosis and treatment in public health facilities was funded by the government. Private health facilities can have this advantage if they are willing to engage with NTP. The public health sector, especially the CHC, does not only provide curative services to TB patients, but also preventive and promotive services. The CHC has the human resources for active case finding, TB contact investigation, and the tracking of TB patients lost to follow-up in the community. This cannot be conducted by private health facilities. Although public sectors, especially the CHC, are the backbone of TB control in Indonesia, the private sector needs to be involved. The number of public health service facilities and their resources are limited, while the private health service facilities are increasing. Most TB patients seek initial treatment at private health care facilities. Networks between the public and private sector have a great opportunity in TB management to reduce missing cases.

In the DPPM strategy, the district health office is responsible for coordinating the network of collaboration between public and private health care facilities, and involving professional organizations in TB control. CHCs coordinate with private physicians, clinics, pharmacies, and laboratories in their working area (sub-district) regarding TB case finding, diagnosis, treatment, contact investigation, logistic, referrals, recording and reporting. All health care facilities are responsible for implementing standardized TB service and mandatory notification [8]. 

One of the problems in DPPM is a readiness to engage. An intervention that can be conducted is the strengthening of the health system through the development of cloud-based information and communication media [10]. Digital health could encourage and strengthen continuing cooperation between and among stakeholders from the public and private sectors. The innovations brought about by digital health are also needed for patient care, referrals, surveillance, e-learning and communication between public and private health services. The Indonesian government advocates digital transformation in health services. This situation has been amplified by the COVID-19 pandemic. Many studies show that the use of digital health that enhance the ease of collaboration working between the public and the private health sectors may help speed up the DPPM implementation [11,12,13].

The existing information system in the TB program is still fragmented, focused on surveillance and has not facilitated all of the private sector, where its existence is increasing. *Sistem Informasi Tuberkulosis Terpadu* (SITT) has been used as a TB information system in Indonesia’s NTP and has been transformed into *Sistem Informasi Tuberkulosis* (SITB) since January 2020 [14]. Currently, SITB could only be accessed by public health services such as CHCs, public hospitals and several private hospitals that collaborate with the NTP, whereas private practitioners, private clinics, private practice midwives, and pharmacies do not have access.

A previous publication mainly focused on the effectiveness and feasibility of the information system that has been built [15,16,17]. A study in Pakistan showed that involving the private sector and laypeople to use a mobile phone application was effective in increasing the TB case notification, as a research in Bandung showed that a mobile phone application for reporting TB-symptomatic individuals and diagnosed TB cases was acceptable and utilized by private practitioners [11,16].

Research in developed countries such as Japan and South Korea shows that public–private partnership in TB control has been well established. Information systems were continuously developed for data transfer, case management, classifying treatment outcome, and contact examination. The national computerized TB surveillance system in Japan was developed to be user-friendly and used to evaluate current TB problems. A web-based TB surveillance system is important for implementing the evidence-based TB control program in Japan. In South Korea, a web-based system launched in 2000. All physicians are required to report a confirmed or suspected TB patient they have diagnosed or treated. Notification data consist of patient identity, examination results, treatments, and treatment outcome. A study showed an increase in the completeness of web-based notifications from 2012 to 2014 in South Korea [18,19].

Studies on the development of the information system based on problems in the field and input from stakeholders in a developing country such as Indonesia will add the current knowledge on how to build effective digital health to strengthen DPPM TB. This study was aimed at exploring the problem that occurs in district-based public–private mix tuberculosis control in the Purwakarta district, and how digital health can help overcome this problem. The purpose of this study is to identify policy problems in DPPM-TB to find solutions and discuss whether digital health can help in their overcoming.

## 2. Materials and Methods

### 2.1. Research Setting

The study was carried out in the Purwakarta district, Java Island, Indonesia. The district has an estimated population of 953,414. The public health services in this district include twenty CHCs called *Puskesmas*, which are spread over seventeen sub-districts and one regional general hospital. Private health services include 57 private physicians, 92 private clinics, and 11 private hospitals.

Digital health has been used in TB management for recording and reporting TB cases through a web-based application called SITB. SITB was centralized and can only be used by health care providers in collaboration with NTP. Indeed, most health care facilities do not or have not collaborated with NTP, while they have great potential in managing TB patients. The lack of concern about the TB problem and institutional support correlated with unreadiness to engage [10]. There is a communication gap between health care facilities and NTP if they only rely on SITB. There is also a WIFI TB application to facilitate private physician reporting TB cases, but this application has not been used in the Purwakarta district. WIFI TB also does not reach potential stakeholders who can contribute to TB management.

### 2.2. Study Design and Sampling Method

This study was a qualitative content analysis research [20,21]. Qualitative research was conducted in order to gain an in-depth understanding of the problem in DPPM and the expectation of the public and the private health sectors of the information system that strengthens DPPM. The research population was stakeholders who come from public and private health facilities. This study applied a purposive sampling technique; informants are selected on the basis of research objectives [22]. All stakeholders were invited to be participants. Then, the stakeholders who were willing to participate were recruited as participants. The researcher succeeded in recruiting 46 informants for in-depth interviews. Then, from the informants, nine people were asked to take part in a focused group discussion. The characteristics of informants can be seen in Table 1.

### 2.3. Data Collection

In-depth interview and focused group discussion (FGD) were conducted form January 2020 to November 2020. Research activities were carried out with health protocols during the COVID-19 pandemic. The interview topics contained: What happened to the public–private partnership in DPPM for TB control? How can digital health enhance public–private partnership? The interview length was 45 min to 1 h and the discussion length was 2 h. In-depth interviews were conducted by 20 trained researchers and research assistants. The results of the in-depth interview analysis were followed up with FGD. The researcher herself was the facilitator for the FGD. The discussion topics contained: submission of core findings from in-depth interview, strengthening of these findings from groups as triangulation, and stakeholder expectations for the development of information system. Secondary data were also obtained from the study of TB program report documents.

### 2.4. Data Analysis

The interviews and discussion were recorded and transcribed into verbatim transcripts. Transcripts were analyzed to obtain codes by the first and second authors (CP and DKS). Code differences were discussed for consensus. After identifying the codes, the same codes were grouped into categories and then the theme was identified. The categories and theme were then discussed and agreed upon by all authors.

Trustworthiness was obtained through triangulation method to compare information between informants. Triangulation method is used to increase the likelihood of credible research result by using several different ways to demonstrate the truth [23]. This study has used several different qualitative data sources, and the information obtained from in-depth interview can be compared with focus group discussion. Researchers have used triangulation of data sources by comparing the responses of informants from the public and the private sectors with various professions and institutions. Peer debriefing was conducted between authors [24].

### 2.5. Ethics

The researcher gave information about the objective and topics to be discussed before conducting in-depth interview and FGD. Informants were asked for their consent and they were aware of the fact that the interviews would be recorded. Ethical approval was obtained from the Padjadjaran University Ethics Committee (112/UN6.KEP/EC/2020).

## 3. Results

The results of this study indicate four main problems that occur in public and private partnerships in tuberculosis care in the Purwakarta district. These problems can be overcome with a health information system, and there are expectations from stakeholders regarding the digital health solution that will be built. Some issues regarding the two topics can be seen in Table 2.

### 3.1. The Problem

#### 3.1.1. Under-Reporting of TB Cases in the Private Health Sector

Table 3 shows that private physician and private clinics have no contribution to TB case notification until 2020. Most of the case notification came from the public health sector.

In the DPPM TB, private clinics and private physicians were under the coordination of the CHC according to the sub-district, where the clinic was located. They should report TB cases to CHC, but this has not happened in the Purwakarta district.

“*All TB cases that I report to SITB were only CHC patients, private clinics in our work area have never reported TB cases to us*.”

(an NTP staff member in a CHC)

#### 3.1.2. Lack of Awareness

Our study found the lack of awareness of private clinic doctors and private physicians in reporting TB cases and how to report them.

“*We didn’t know that TB cases had to be reported by name by address, I only report the top ten disease in my clinic to CHC*.”

(a doctor in private clinic)

“*I only examine patients, if anyone is diagnosed with TB, I write it down in the medical record. I don’t know about TB case recording and reporting*.”

(a doctor in private clinic)

“*We have never known or used a WIFI TB application*.”

(private physician)

#### 3.1.3. Lack of Communication

Lack of communication has occurred between health workers in the TB patient referral process. This condition has caused problems in recording, reporting, and treating patients. Lack of communication between private practitioners and CHCs for contact investigation causes many undiagnosed TB cases (missing cases).

“*Referral patients with incomplete data will be a problem in reporting system, I gave the medicine but I didn’t enter patient data into SITB*.”

(an NTP staff member in a CHC)

“*Patient referrals are not accompanied by clear information about treatment categories, history of examinations and medications that were given to patients, it makes us confused to continue patient’s treatment*”

(an NTP staff member in a CHC)

“*I have a child patient with TB meningitis, there must be adult TB patients around the child. Who is in charge of conducting contact investigation? I can’t do it*.”

(a pediatrician)

#### 3.1.4. Limitation of Existing Information System

The current use of the existing information system in the TB program called SITB is focused on recording and reporting TB cases, and access is limited to the public sector and several private sector bodies that already have MoU with the district health office, while private practitioners and the private clinic in the Purwakarta district do not have access.

“*Currently SITB can only be accessed by regional general hospital, 20 CHCs, and 5 private hospitals that already have an MoU with NTP*.”

(an NTP staff member in DHO)

“*Yes, I have heard about the WIFI TB application, but I can’t see TB patient data reported by private doctors*.”

(an NTP staff member in DHO)

Based on understanding, meaning, and interpreting as a qualitative analysis process, the utilization of SITB is presented in Table 4.

### 3.2. Expectations of Digital Health

#### 3.2.1. Ethical Issues

The security of patient data must be ensured in digital health. TB patient data reported by private practitioners through application should only be accessed by an authorized person.

“*TB patient data should not be open to all application users, patient can be angry and embarrassed*.”

(a doctor in CHC)

“*My cadre and I once conducted a contacts investigation around the TB patient’s house. We asked the head of the neighborhood for permission. But the patient became angry because the head of the neighborhood knew his illness*.”

(an NTP staff member in a CHC)

#### 3.2.2. Avoid Redundancy

Health workers hope that they do not have to do paper-based TB case reporting. They also hope that TB case reporting only needs to be conducted through one information system.

“*I prefer using the application for TB case reporting, paper-based reporting is a hassle for me. I hope I don’t have to report using paper anymore*.”

(private physician)

“*Can the new information system be connected to SITB? Lest we have to repeatedly input TB patient data*.”

(a doctor in CHC)

#### 3.2.3. User-Friendly

Private practitioners must report TB cases to the CHC in their sub-district. The use of digital health is expected to make the reporting process easier; private practitioners do not need to come to the CHC.

“*I hope the application is easy to use by all ages, both young and old, because many health workers are old*.”

(a doctor in public hospital)

“*I don’t have time to come to CHC, I’m too busy taking care of patients. If there is an application, I can easily report TB cases*.”

(a doctor in private clinic)

#### 3.2.4. Intervention Package

The use of digital health requires a commitment from all stakeholders in DPPM. TB program training and memorandum of understanding (MoU) are needed to strengthen the commitment of the private sector.

“*There needs to be an understanding between private and public health sectors otherwise any program will not work no matter how good it is*.”

(an NTP staff member in DHO)

“*Private doctors need to be explained about their role in the TB program and there needs a MoU to strengthen their commitment*.”

(an NTP staff member in a CHC)

## 4. Discussion

Our study highlighted the lack of communication between the public and the private health sectors in tuberculosis control as a major problem in DPPM TB. Digital health is needed to facilitate communication between all stakeholder in DPPM tuberculosis and to fill the gap in the existing tuberculosis information system. One of the key domains of private sector engagement is information exchange between the public and the private health sectors [25].

Lack of communication has occurred between public and private health workers in our study site. This happens in TB patient’s referral. Some private doctors did not use standardized TB patient referral forms; they only use general patient referral letters or even prescription papers to refer TB patients with incomplete information. The doctor must provide information about the patient’s identity, diagnosis, case type, examination and treatment history. Incomplete information caused confusion for health workers who received patient referrals, and because of this, an error occurred in the patient treatment. In the referral process, the health worker could not confirm whether the patient came to the referral health facility or not, meaning the patient could not come and continue his treatment. A descriptive questionnaire-based study in South Africa found that the inability to follow up was the most important reason for the failure of referral of MDR and XDR-TB patients to specialist care, both for hospital- and CHC-diagnosed cases. The most operational problem was the lack of communication procedures regarding referral [26].

Lack of communication also has an impact on TB case finding. Private doctors have known that contact investigations are needed when they find TB cases, but they do not have the time and resources. Communication between private practitioners and CHCs is needed for contact investigation because only CHCs can do this. Contact investigation can improve TB case finding. Stopping TB transmission requires finding all cases promptly and treating them until healed [27]. Private practitioners need to report TB patient data to CHCs so that CHCs can carry out contact investigations.

Communication between public and private health sectors has not been accommodated by the existing information system. Limitation of the existing information system was one of the causes of missing tuberculosis cases. SITB is focused on recording and reporting TB cases. Communication is important in delivering information about the TB program, patient referrals, and contact investigation; therefore, a communication and information system is required to facilitate the flow of information and communication between the public and the private health sectors.

SITB access is limited to the public sector and several private sector bodies, whereas most patients come to private health facilities. Pharmacies are places where TB patients seek initial treatment when experiencing TB symptoms; they have the potential to find and communicate suspected TB cases to the CHC [5]. Laboratory workers, midwives, and health cadres can also find a suspected TB patient in their service activities, so they also need access to digital health.

The public and the private sectors have agreed on the importance of digital health to support DPPM TB. They have some expectations of the information system to be built. The security of patient data must be ensured in digital health. Most TB patients become angry or embarrassed when others find out about their illness. Patient diagnosis is a medical secret; there were ethical concerns among private practitioners about patient privacy, especially considering that there is a stigma about TB in the community [28]. TB patient data reported by private practitioners through application should only be accessed by the CHC for case reporting to SITB and for contact investigation. Each CHC has a working area at the sub-district level; they can only access patient data living in their working area.

Private practitioners hope that the data to be entered in the application should not be too much; the application can be used easily and instructions on the application must be clear and use Bahasa to make it easier to use. They also hope that they do not have to report repeatedly using paper or some information system. They were already busy taking care of patients. Users’ adoption and use of a technology is predicted by users’ perception about ease of use [29].

The use of digital health requires a commitment from all stakeholders in DPPM. It is important to provide understanding to the private sector of their role in the TB program and the importance of collaboration between public and private health sectors in TB control. Our study found a lack of awareness among private clinic doctors and private physicians in reporting TB cases and how to report them. A study in Yogyakarta found that private practitioners could not notify TB cases due to a lack of policy knowledge [30]. Other studys in Chennai found that private practitioners still fail to notify TB cases even though they were aware of mandatory notification [31]. The private sector staff need training to raise awareness of the importance of their role in DPPM TB. This issue can be explained at the time of training the use of application. Regulation and MoU are needed to strengthen commitment [32].

This study is qualitative research that explores a deep understanding of the real problems that occur in the implementation of DPPM-TB in the field. The results of this study support the strengthening of the building blocks of the local health system. The generalizability of the findings to other DPPM situations in another district should be made with caution and will depend on consideration of the context.

## 5. Conclusions

The lack of communication between the public and the private health sectors in TB control was a major problem in DPPM TB in our study site. DPPM TB needs to be strengthened with a digital health solution to ensure the flow of information and communication between the public and the private health sector, and to overcome the limitations of existing information systems. Digital health development needs to be adapted to conditions in the field. The use of digital health requires a commitment from all stakeholders in DPPM, so that an intervention package is needed to increase their awareness and commitment. Further research is needed to generalize the result of this qualitative research by conducting quantitative research.

## Figures and Tables

**Table 1:** Characteristic of informants.

		In-Depth Interview		Focus Group Discussion	
		N	Percentage (%)	n	Percentage (%)
Gender	Male	12	26.1	1	11.1
	Female	34	73.9	8	88.9
Profession	Doctor	34	74	7	77.7
	Nurse/midwife	6	13	-	-
	NTP staff	6	13	2	22.3
Sector	Public	23	50	4	44.4
	Private	23	50	5	55.6
Institution	CHC	20	43.5	4	44.4
	Private physician	10	21.7	2	22.2
	Private clinic	8	17.4	2	22.2
	District Health Office	8	17.4	1	11.2

**Table 2:** Issues related to DPPM TB problems and expectations of digital health.

Category	Source of Information
**The problem**	
− Under-reporting of TB cases in the private health sector	NTP staff (6 persons) Private physician (3 persons) Private clinic (2 persons)
− Lack of awareness	NTP staff (6 persons) Private physician (4 persons) Private clinic (4 persons)
− Lack of communication	NTP staff (6 persons) Private physician (4 persons) Private clinic (3 persons)
− Limitation of existing information system	NTP staff (6 persons) Private physician (4 persons) Private clinic (2 persons)
**Expectation of digital health**	
− Ethical issues	NTP staff (2 persons) Doctor in CHC (2 persons) Private physician (1 person)
− Avoid redundancy	NTP staff (2 persons) Doctor in CHC (2 persons) Private clinic (1 person)
− User-friendly	Doctor in CHC (1 person) Private physician (2 persons)
− Intervention package	NTP staff (2 persons)

**Table 3:** TB case notification in Purwakarta district.

Health Care Facilities	2018	2019	2020
**Public**			
Community health centres	1073	1120	1012
Public hospital	103	294	254
**Private**			
Private hospital	0	735	353
Private clinic and private physician	0	0	0
Total	1176	2149	1619

**Table 4:** Utilization of SITB by stakeholders in DPPM.

Stakeholder	Public	Private
Primary health care	All CHC have used (20 CHC)	No one has access yet (57 private physician and 92 private clinics)
Secondary health care	Regional general hospital has used (the only one hospital)	Only 5 from 11 hospital have used
Pharmacy	Already have access but have not used it (20 units public pharmacies at CHC and 1 unit at regional general hospital)	No one has access yet
Laboratory	All laboratories have used (20 units public laboratory at CHC and 1 unit at regional general hospital)	No one has access yet
Cadre	-	No one has access yet

## Data Availability

The datasets used and analyzed during the current study are available from the corresponding author on reasonable request.

## Abbreviations

CHC	Community health center
DPPM	District-based public–private mix
FGD	Focused group discussion
GP	General practitioner
MDR-TB	Multidrug-resistant tuberculosis
MoU	Memorandum of understanding
NTP	National tuberculosis control program
SITB	*Sistem informasi tuberkulosis*
SITT	*Sistem informasi tuberkulosis terpadu*
TB	Tuberculosis
XDR-TB	Extensively drug-resistant tuberculosis
